# Predictors of Successful First-Pass Thrombectomy with a Balloon Guide Catheter: Results of a Decision Tree Analysis

**DOI:** 10.1007/s12975-020-00784-2

**Published:** 2020-05-23

**Authors:** Aglaé Velasco Gonzalez, Dennis Görlich, Boris Buerke, Nico Münnich, Cristina Sauerland, Thilo Rusche, Andreas Faldum, Walter Heindel

**Affiliations:** 1 Department of Clinical Radiology, Institute of Clinical Radiology and Neuroradiology, University Hospital of Muenster, Albert-Schweitzer-Campus 1, Building A1, 48149 Muenster, Germany; 2 Institute of Biostatistics and Clinical Research, University of Muenster, Schmeddingstraße 56, 48149 Muenster, Germany

**Keywords:** Stroke, Thrombectomy, Suction, Carotid arteries, Circle of Willis

## Abstract

Complete recanalization after a single retrieval maneuver is an interventional goal in acute ischemic stroke and an independent factor for good clinical outcome. Anatomical biomarkers for predicting clot removal difficulties have not been comprehensively analyzed and await unused. We retrospectively evaluated 200 consecutive patients who suffered acute stroke and occlusion of the anterior circulation and were treated with mechanical thrombectomy through a balloon guide catheter (BGC). The primary objective was to evaluate the influence of carotid tortuosity and BGC positioning on the one-pass Modified Thrombolysis in Cerebral Infarction Scale (mTICI) 3 rate, and secondarily, the influence of communicating arteries on the angiographic results. After the first-pass mTICI 3, recanalization fell from 51 to 13%. The regression models and decision tree (supervised machine learning) results concurred: carotid tortuosity was the main constraint on efficacy, reducing the likelihood of mTICI 3 after one pass to 30%. BGC positioning was relevant only in carotid arteries without elongation: BGCs located in the distal internal carotid artery (ICA) had a 70% probability of complete recanalization after one pass, dropping to 43% if located in the proximal ICA. These findings demonstrate that first-pass mTICI 3 is influenced by anatomical and interventional factors capable of being anticipated, enabling the BGC technique to be adapted to patient’s anatomy to enhance effectivity.

**Electronic supplementary material:**

The online version of this article (10.1007/s12975-020-00784-2) contains supplementary material, which is available to authorized users.

## Introduction

In acute ischemic stroke, clinical outcomes depend heavily on rapid and complete recanalization [1–4]. The goal for interventional therapies is thus complete recanalization after a single retrieval maneuver, which is an independent factor for good clinical outcome [5]. Combining stent retrievers (SRs) and balloon guide catheters (BGCs) is a commonly used endovascular clot removal technique [6]. This mechanical thrombectomy (MT) technique requires antegrade flow to be arrested (temporal occlusion of the carotid artery) and simultaneous aspiration through the BGC for retrieval (flow reversal) [7–9]. Unfortunately, the chances of achieving Modified Thrombolysis in Cerebral Infarction Scale (mTICI) 3 decrease with each retrieval maneuver [5, 10, 11]. Consequently, it is essential to analyze the anatomical and angiographic factors that could alter the effectiveness of clot removal on the first attempt using the combined SR-BGC technique. These biomarkers indicative of the difficulty of clot removal could be identified prior to therapy, thereby enabling the mechanical thrombectomy technique to be adapted to each patient’s anatomical conditions.

Few studies have evaluated the effect of angiographic factors such as carotid tortuosity on recanalization rates, with marked disparity in their results. Yilmaz et al. [12] used computed tomography angiography (CTA) to study the influence of carotid elongation on recanalization (mTICI ≥ 2b independent of the number of passes) in a series of 54 MTs but failed to show any impact of elongation on the angiographic results. In contrast, more recently, Jeong et al. [13] examined the frequency of successful recanalization (mTICI ≥ 2b) in the presence of carotid tortuosity and BGC placement for therapy (distally versus proximally in the internal carotid artery (ICA)), but they did not provide any related statistical test results. Although the rate of successful recanalization was higher in the group with the combination of “tortuosity absent” and distal BGC positioning in the ICA, the published data do not provide statistical confirmation that carotid elongation significantly influences BGC positioning or significantly affects the rate of successful recanalization [13]. To the best of our knowledge, the effect of carotid tortuosity on the position of the BGC for treatment and on recanalization rates has not yet been determined. Thus, greater insight into the relationships among all these factors is required, focusing on the best angiographic outcome attainable, complete recanalization (mTICI 3) after one SR pass.

A further matter to consider is whether the presence of communicating arteries could influence MT outcomes. In normal circumstances, the middle cerebral artery (MCA) and the anterior cerebral artery (ACA) are supplied mainly by the ICA. However, in cases of acute occlusion, flow direction and volume through the communicating arteries may change in response to the new cerebral perfusion requirements [14–16]. Reversal of communicating artery flow to the occlusion side results in a supplementary continuous blood supply that should also be aspirated through the BGC. Thus, the suction effect from aspiration through the BGC under conditions of flow arrest in the ICA is enhanced when there are no communicating arteries and could hypothetically decrease when these arteries are present [7].

The objective of this analysis was to assess the influence of carotid elongation, BGC positioning, and anatomical variations of the circle of Willis (CoW) on complete recanalization rates after one pass in 200 consecutive patients who underwent mechanical thrombectomy with BGC. Additionally, the National Institutes of Health Stroke Scale (NIHSS) scores were correlated with the angiographic findings.

## Methods

### General

The primary objective of this study was to assess the association between carotid elongation and BGC location for therapy and its impact on one-pass complete recanalization. Secondarily, the presence of communicating arteries ipsilateral to the stroke side and their influence on the angiographic results and clot migration during therapy were also analyzed. This retrospective study was approved by our institution’s review board. Informed consent was waived. This study received no industry support.

### Patient Selection

Between January 2016 and December 2018, 283 patients were treated consecutively for acute ischemic stroke of the anterior circulation by MT with SR through a BGC as the first-choice technique, in all cases using flow arrest and continuous manual aspiration during the retrieval maneuver. Patients from this group with tandem stenosis/occlusions (*n* = 47) or clot extension into the extracranial carotid artery (*n* = 36) were excluded, resulting in a total study group of 200 patients for this retrospective analysis.

### Assessment of the Results

We retrospectively compiled and analyzed the clinical patient data, including stroke risk factors, stroke demographics, and prior treatment with intravenous tissue plasminogen activator (tPA). NIHSS scores at presentation and at discharge were obtained from Neurology Department records. One NIHSS score at presentation and six scores at discharge went unrecorded. In addition, 33 patients died while in hospital; their final NIHSS scores were not documented.

**Fig. 1:**
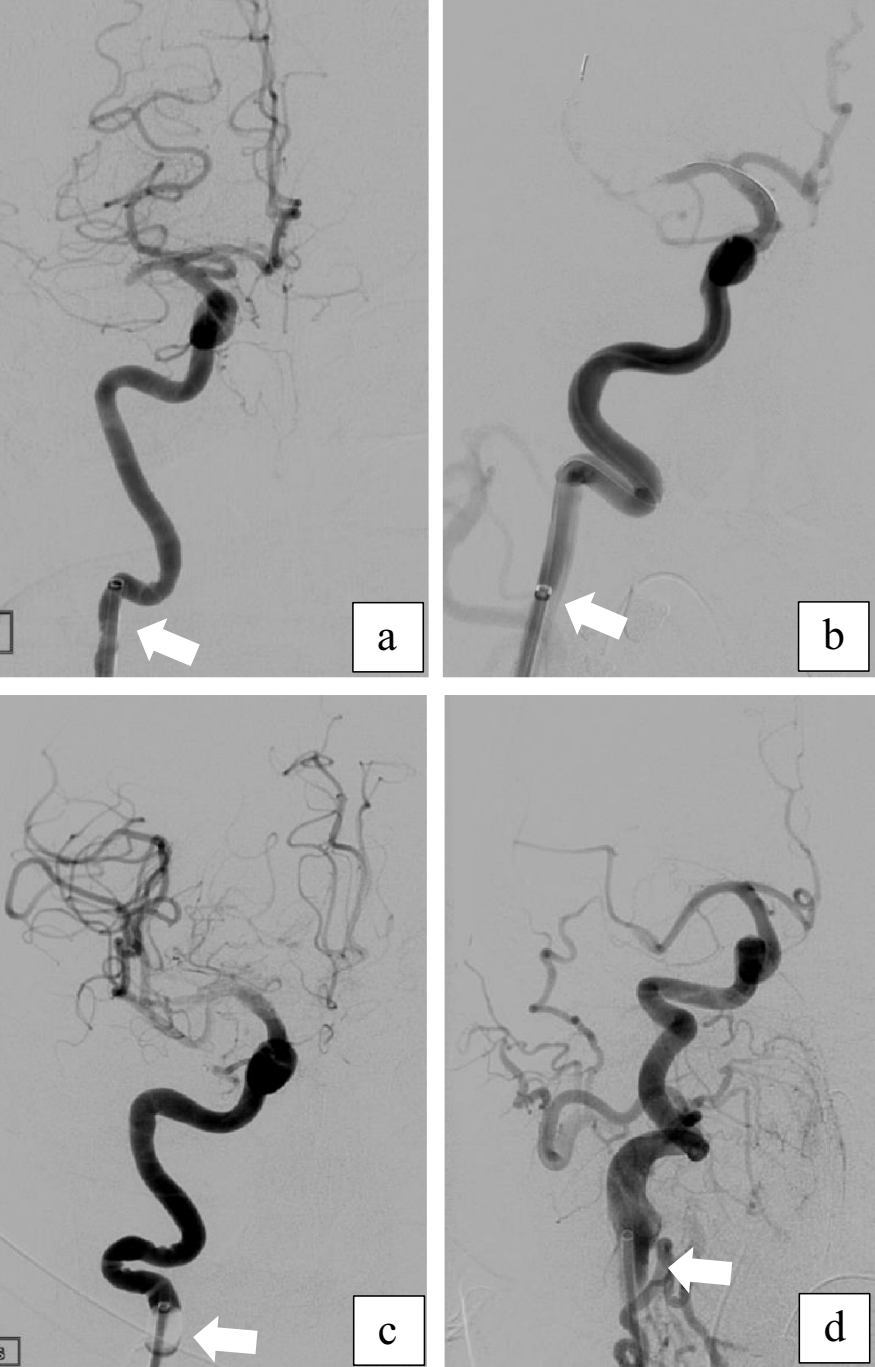
**a**–**d** Frontal and oblique views of carotid elongation using digital subtraction angiography (DSA). Four examples of internal carotid artery (ICA) elongation distal to the tip of the balloon guide catheter (BGC). In all these cases, the BGC was placed in the proximal ICA (dividing the ICA into three segments from the subpetrosal segment to the extracranial bifurcation, the proximal ICA is the caudal two thirds of the cervical carotid). Case A shows occlusion of the middle cerebral artery and a single 90° kink. Case B shows double ICA kinking and an open retriever device in the distal M1 segment into the M2 segment through a distal M1–M2 clot. Cases C and D depict multiple ICA kinks (the follow-up angiography after recanalization in case C was inadvertently performed under balloon inflation)

**Fig. 2:**
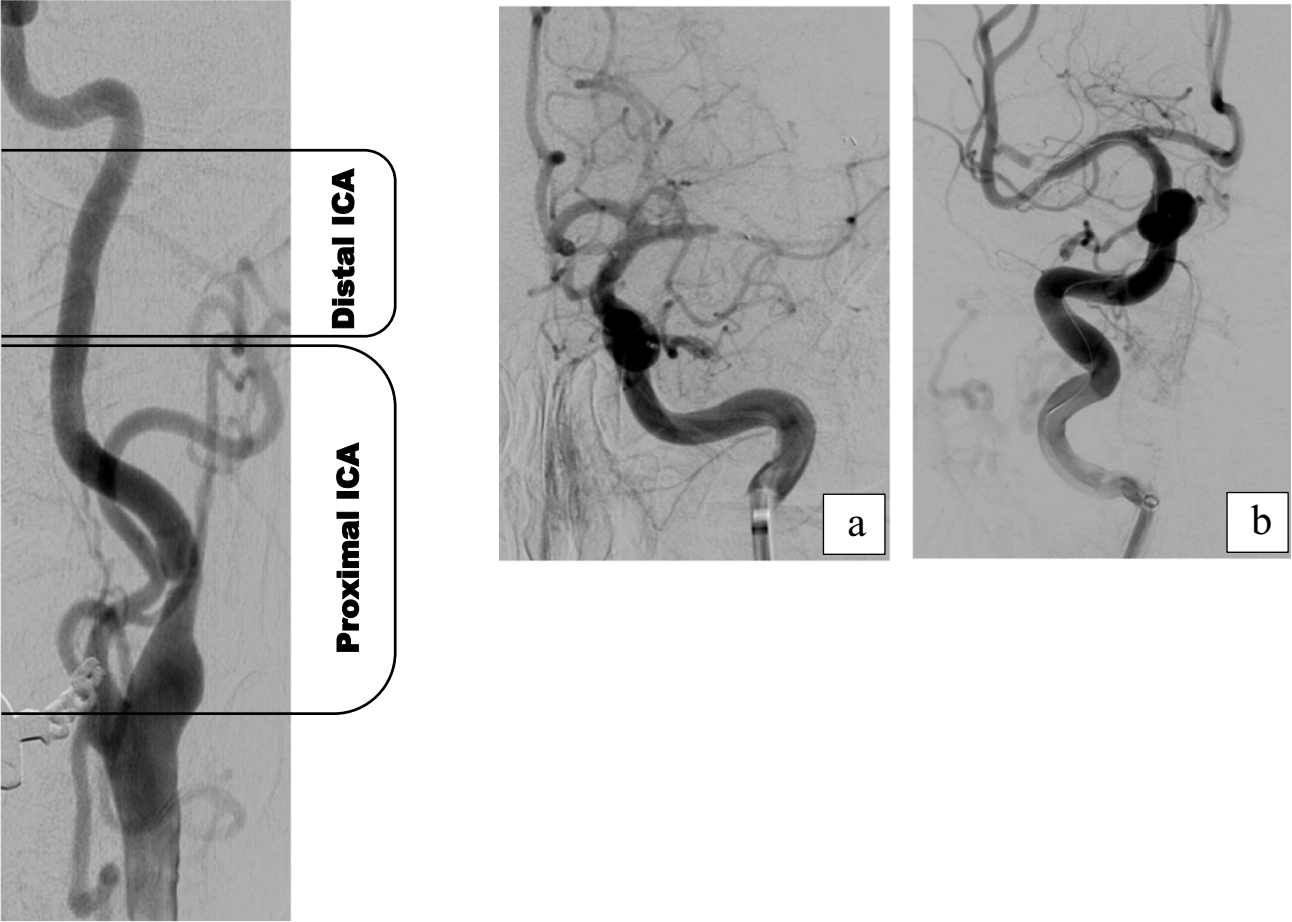
Balloon guide catheter position (BGC) in the carotid artery. From right to left, angiographic classification of BGC location for treatment: dividing the internal carotid artery (ICA) into three segments from the subpetrosal segment to the extracranial bifurcation, the proximal ICA is the caudal two thirds of the cervical carotid. The following two images show distal BGC positioning (**a**) and proximal BGC positioning (**b**) in the ICA

The anatomy of the CoW ipsilateral to the stroke side was determined from the invasive angiographic images on record and correlated with the features of the CTA images to obtain a final evaluation. Variations in diameters between the MCA and the ICA were determined as the ratio of the diameter of the ICA (measured distal to the posterior communicating artery (PCoA) segment) to that of the proximal M1 segment. Elongation of the carotid artery was defined on the basis of such angiographic features as the presence of kinking or tortuosity of the ICA distal to the position of the BGC (Fig. 1), as proposed by Jeong et al. [13]. BGC positioning for treatment was classified into three groups based on dividing the ICA into three segments: (1) the distal ICA (BGC tip located in the distal third of the ICA, i.e., subpetrosal placement), (2) the proximal ICA (BGC tip located in the caudal two thirds of the ICA), and (3) the distal common carotid artery (CCA) (Fig. 2).

All patients were treated using an SR + microcatheter through an 8-French BGC (flow arrest + manual aspiration during retrieval) as the first-choice technique. Selection of the SR device was left to the operator. The procedure time, defined as the time elapsing between the first angiographic image obtained using a guide catheter in the extracranial carotid artery and the end of SR recanalization, was also recorded [8]. The degree of postintervention vessel recanalization was based on the mTICI score, whereby grade 2b indicates at least 50% reperfusion of the affected region and grade 3 complete reperfusion. For the purpose of this analysis, cases of mTICI grade 2c were included in the mTICI 3 population. Complete recanalization after a single retrieval maneuver (first-pass effect) [5] was defined as achieving mTICI 3 after a single pass with the SR. Successful revascularization was defined as a final mTICI grade of at least 2b on conclusion of the procedure after up to three passes. Postinterventional mTICI grades were assigned by the treating physician at the end of the intervention in the clinical setting. The grades were re-evaluated retrospectively by a second neuroradiologist (AVG) to obtain a final assessment; discrepancies were settled by consensus. Any suspicion of clot migration during therapy necessitated retrospective evaluation of the CTA to rule out multiple emboli in the distal territories at presentation.

Follow-up imaging (CT) was performed after 24 h. Adverse procedure-related events were recorded. Hemorrhage was graded according to the method used in the European Cooperative Acute Stroke Trials [17].

### Statistical Analysis

Three of the authors were involved in the statistical analysis (AVG (neuroradiologist), DG (statistician), and CS (statistician)). The Mann-Whitney *U* test, chi-square test, and Fisher’s exact test were used to compare the continuous and categorical baseline variables between the first-pass mTICI 3 groups (yes/others). Changes in the initial and final NIHSS scores were compared using the Wilcoxon signed-rank test.

The association between carotid elongation (yes/no) and BGC position was evaluated using the chi-square test. Additionally, the association between elongation and BCG position with first-pass mTICI 3 and successful recanalization was analyzed using univariate and multivariable logistic regression models while adjusting for stroke laterality, intravenous thrombolysis, previous anticoagulation, ICA/MCA ratio, intervention duration, and ipsilateral complete CoW. Wald test *p* values were recorded together with the odds ratio (OR) (95% confidence intervals (CIs)). *p* values ≤ 0.05 were deemed to indicate a statistically significant difference. All reported *p* values were two-sided. Finally, a decision tree was implemented to predict one-pass complete recanalization based on all the variables, according to the following settings: maximum depth of 4, minimum splitting size of 10, minimum child size of 5, 10-fold cross-validation, alpha of 0.05, and splitmerged. This analysis was Bonferroni adjusted. SPSS (version 25; IBM, Armonk, NY, USA) was used for all statistical analyses.

## Results

### Demographics

This study included 115 women (mean age, 78 ± 12 years; age range, 31–96) and 85 men (mean age, 70 ± 15 years, age range, 32–96) (*p* < 0.0001). The baseline characteristics of the 200 patients in whom complete recanalization (mTICI 3) was achieved with a single retrieval maneuver are presented in Table 1. The median initial NIHSS score was 15 (interquartile range (IQR) 11–17), decreasing significantly at discharge (median final NIHSS score (IQR) 3 (2–7); *p* < 0.0001).

**Table 1:** Patient demographics, occlusions, and interventions

	Total (*n* = 200)	Complete one-pass recanalization		*p* value
		Yes (*n* = 102)	No (*n* = 98)	
Gender, female, *n* (%)	115	57 (55.9)	58 (59.2)	0.0162
Age, years (IQR)	77 (70, 84)	76 (68, 81)	78 (71, 85)	0.678
Hypertension, *n* (%)	120 (60)	55 (53.9)	65 (66.3)	0.073
Diabetes mellitus, *n* (%)	35 (17.5)	21 (20.6)	14 (13.7)	0.241
Smoker, *n* (%)	24 (12)	14 (13.7)	10 (10.2)	0.444
Atrial fibrillation, *n* (%)				
New diagnosis	49 (24.5)	28 (27.5)	21 (21.4)	0.536
Previous diagnosis	50 (25)	26 (25.5)	24 (24.5)	
Previous anticoagulation therapy, *n* (%)	85 (42.5)	46 (45.1)	39 (39.8)	0.048
Dyslipidemia, *n* (%)	42 (21)	21 (21.4)	21 (21.4)	0.884
Stroke demographics				
Stroke laterality, *n* (%)				
Right	102 (51)	60 (58.8)	42 (41.2)	0.024
Left	98 (49)	42 (42.9)	56 (57.1)	
IV thrombolysis	109 (54.5)	55 (53.9)	54 (55.1)	0.867
Location of the occlusion, *n* (%)				
ICA	31 (15.5)	17 (16.7)	14 (14.3)	0.201
Proximal M1^†^	67 (33.5)	38 (37.3)	29 (29.6)	
Distal M1^†^	65 (32.5)	32 (31.4)	33 (33.7)	
Proximal M2^‡^	29 (14.5)	14 (13.7)	15 (15.3)	
Distal M2^‡^	8 (4)	7 (7.1)	1 (1)	
Initial median NIHSS score (IQR)	15 (11–17)	15 (11–17)	15 (11–17)	0.966
NA	1	1	–	
Final median NIHSS score^$^ (IQR)	3 (2–7)	3 (2–7)	4 (2–7)	0.379
NA^†^	39	15	24	
Final NIHSS score − initial NIHSS score				
Median (IQR)	9 (5–13)	9 (5–13)	10 (5–12)	0.795
NA	40	16	24	
In-hospital mortality, *n* (%)	42 (21)	17 (16.7)	25 (25.5)	0.164

In the statistical test, the chi-square test was used for categorical variables and the Mann-Whitney test for ordinal variables. Data have been expressed as median values (interquartile range (IQR)) or numbers (no.) (percentage (%))

*ICA* internal carotid artery, *NIHSS* National Institutes of Health Stroke Scale

^†^M1 segment of the middle cerebral artery (MCA)

^‡^M2 segment of the MCA

^$^Final NIHSS score was not available (NA) for 39 patients, including 33 deaths during hospitalization, and was unrecorded in 6 cases. Units in parentheses are percentages or ranges; error is expressed as ± SD

Several types of SR were used, most frequently the preset 4 (*n* = 144) (Preset SR; Phenox, Bochum, Germany) and the preset 6 (*n* = 37). In most cases, a single SR was used for thrombectomy (85%, 170/200). Multiple SRs (15%, 30/200) were associated with significantly longer intervention times (median duration of MT with multiple SRs, IQR 60 min (40–80) versus 17 min (14–26) with a single SR; *p* < 0.0001) (see the supplemental material, Table 1: types of SRs used).

In the non-enhanced follow-up CT scan after 24 h, hemorrhagic complications with mass effect (parenchymal hematoma type 2 (PH-2)) were observed in six cases (3%), hemorrhagic complications without mass effect (parenchymal hematoma type 1 (PH-1)) in 21 cases (10.5%), and subarachnoid hemorrhage in 20 cases (10%). The inpatient mortality rate was 21% (41/200). The logistic regression showed that in-hospital mortality in the elderly was higher than that in younger patients, with the risk increasing by a factor of 1.034 (1.004–1.066) per year (*p* = 0.028).

### Per-Pass Achievement of mTICI 3

**Fig. 3:**
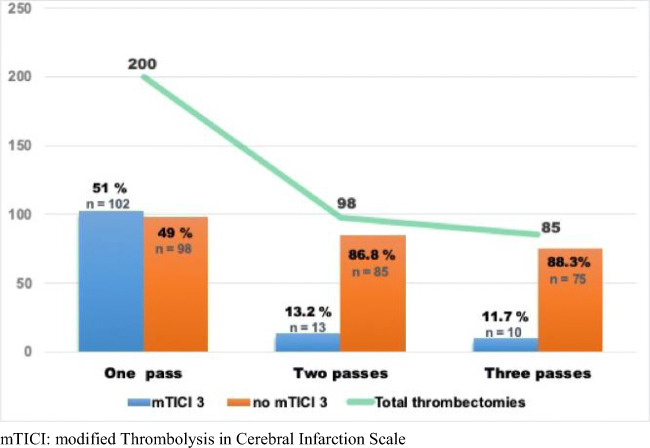
Changes in mTICI 3 rates per stent retriever pass

Recanalization was successful (mTICI 3/2b after up to three passes) in 83.5% of cases (167/200). Irrespective of the number of passes, mTICI 3 was achieved in 62.5% of cases (125/200). In 102 of 200 cases (51%), complete recanalization was achieved with one pass. Figure 3 summarizes the angiographic mTICI 3 rates achieved per pass. The chances of mTICI 3 were reduced to one fifth after the first pass.

### Elongation and BGC Positioning

Positioning of the BGC for therapy depended on the presence or absence of carotid elongation (*p* < 0.0001). The absence of carotid elongation increased the likelihood of the BGC being placed more distally in the ICA and vice versa. Rates of carotid elongation with respect to the position of the BGC for therapy are set out in Table 2.

**Table 2:** Carotid artery tortuosity and BGC positioning for mechanical thrombectomy

Balloon guide catheter Location	Total (*n* = 200), *n* (%)	Carotid artery tortuosity		*p* value
		Yes (*n* = 83), *n* (%)	No (*n* = 117), *n* (%)	
Distal ICA	109 (54.5)	15 (18.1)	94 (80.3)	< 0.0001
Proximal ICA	82 (41)	59 (71.1)	23 (19.7)	
Distal common carotid artery	9 (4.5)	9 (10.8)	0	

Two-sided Fisher’s exact test

*ICA* internal carotid artery

According to the univariate analysis, one-pass complete recanalization depended significantly on the presence of carotid elongation and the BGC position (see Table 3). However, according to the multivariate model, carotid elongation was the only significant factor associated with mTICI 3 after a single pass. A straight carotid artery (no elongation) increased the likelihood of complete recanalization in one pass by a factor of 2.410 (95% CI, 1.138–5.104; *p* = 0.0216). Table 4 outlines the effect of BGC positioning, carotid elongation, lateralization and location of the occlusion, and intravenous thrombolysis on complete recanalization after one pass.

**Table 3:** Univariate analysis of anatomical and interventional characteristics in cases of complete one-pass recanalization

	Complete one-pass recanalization			*p* value
	Total (*n* = 200)	Yes (*n* = 102)	No (*n* = 98)	
Retrieval conditions				
Ipsilateral ACoA and PCoA, *n* (%)	45 (22.5)	18 (40)	27 (60)	0.170
ICA/MCA ratio				
Median (IQR)	1.4 (1.3, 1.5)	1.4 (1.3, 1.5)	1.4 (1.3, 1.56)	0.678
NA	12	8	4	
Elongation, *n* (%)	83 (41.5)	27 (26.5)	56 (57.1)	< 0.0001
Intervention characteristics				
Duration of intervention (min), median value (IQR)	19 (15–36)	16 (14–18)	35 (22–59)	< 0.0005
BGC position, *n* (%)				
Distal ICA	109 (54.5)	70 (68.6)	39 (39.8)	< 0.0001
Proximal ICA	82 (41)	31 (30.4)	51 (52)	
Distal common carotid artery	9 (4.5)	1 (1)	8 (8.2)	
Migration to new territory, *n* (%)	6 (3)	2 (2)	4 (4.1)	0.379

In the statistical test, the chi-square test was used for categorical variables and the Mann-Whitney test for ordinal variables. Data have been expressed as median values (interquartile range (IQR) or numbers (*n*) (percentage (%))

*ACoA* anterior communicating artery, *PCoA* posterior communicating artery, *ICA* internal carotid artery, *MCA* middle cerebral artery

**Table 4:** Multiple logistic regression for predicting complete one-pass recanalization in mechanical thrombectomies through a balloon guide catheter (BGC)

	OR (95% CI)	*p* value
BGC position		
Distal ICA	1.903 (0.913–3.967)	0.086
Common carotid artery	0.352 (0.040–3.076)	0.352
Proximal ICA (ref)	–	
Carotid elongation		
Yes	0.415 (0.196–0.879)	0.022
No (ref)	–	
Lateralization of occlusion		
Right	1.860 (1.011–3.423)	0.046
Left (ref)	–	
Location of occlusion		
ICA	1.103 (0.479–2.543)	0.817
MCA (ref)	–	
IV thrombolysis		
Yes	1.039 (0.564–1.915)	0.902
No (ref)	–	

*ICA* internal carotid artery, *MCA* middle cerebral artery, *CI* confidence interval

To estimate the effect of BGC position, we performed a subgroup analysis on the thrombectomies without carotid elongation (*n* = 117). Distal placement of the BGC in the ICA was an independent factor for complete recanalization after a single SR pass (reference: proximal ICA location; OR 3.188; 95% CI, 1.163–8.740; *p* = 0.024), as was the laterality of the occlusion (reference: left side; OR 2.619; 95% CI, 1.151–5.956; *p* = 0.022).

The best exploratory conditions for achieving one-pass mTICI 3 based on the interaction term were as follows: BGC in the distal ICA + no elongation (68% probability), followed by BGC in the proximal ICA + no elongation (45%); BGC in the proximal ICA + elongation (45%); BGC in the distal ICA + elongation (33%); and BGC in the CCA + elongation (16%).

#### Decision Tree Analysis

**Fig. 4:**
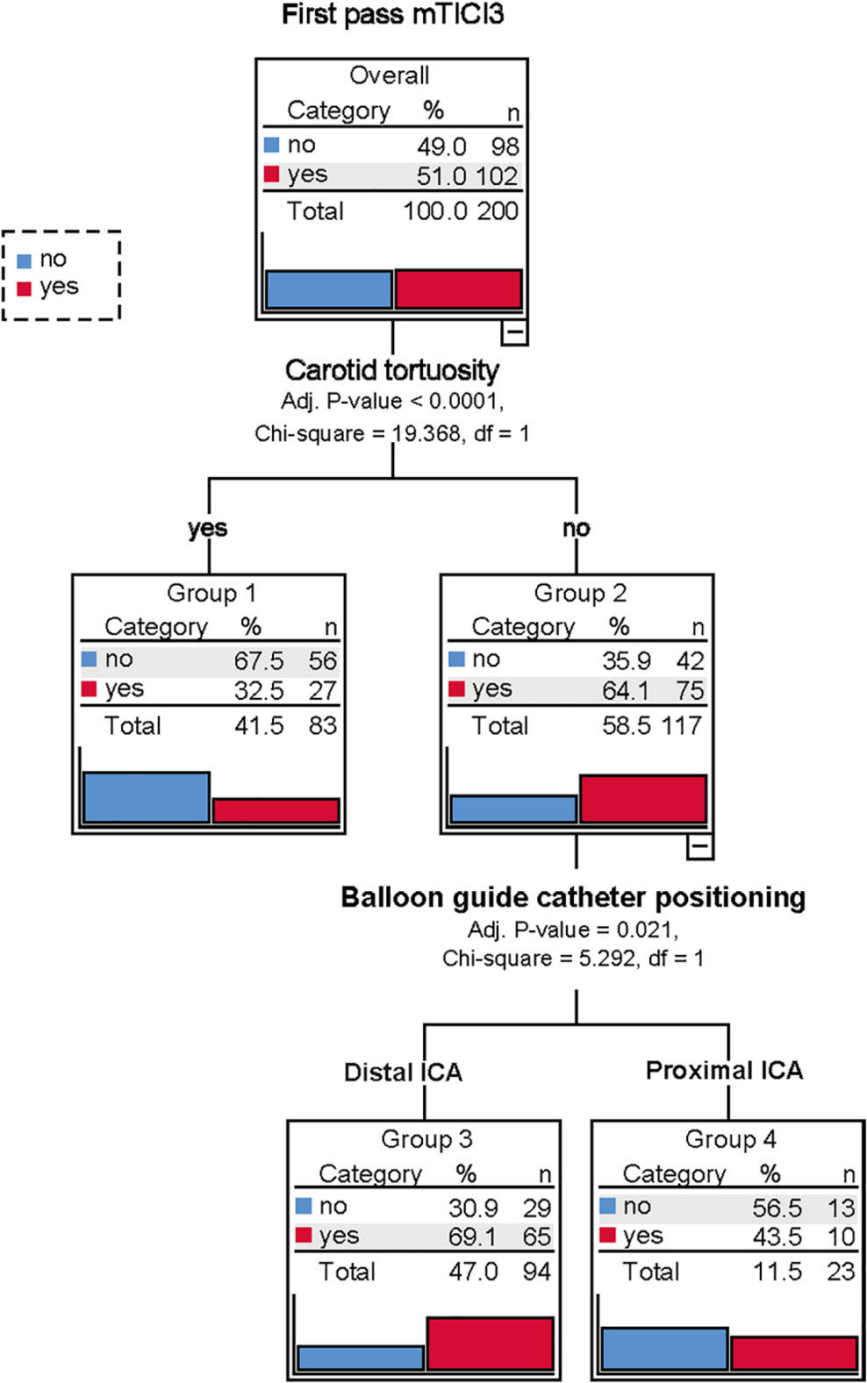
Decision tree for predicting complete one-pass recanalization

To verify the findings, a machine learning decision tree was developed to ascertain the relevant variables for predicting complete recanalization after the first pass. In addition, the decision tree could potentially enable further refinement of patient classification, as appropriate, by adding other variables, always maintaining a minimum significance level of 0.05 between subgroups of patients. As depicted in Fig. 4, the decision tree confirms and summarizes the abovementioned results of the regression analysis.

Carotid tortuosity was selected as the first variable for predicting complete recanalization after one pass. In the presence of carotid elongation, the likelihood of one-pass complete recanalization was 32.5% (*p* < 0.0001). If the carotid artery was straight, the position of the BGC in the ICA was the most important factor for predicting the outcome. If the BGC was positioned in the distal ICA, the probability of complete recanalization from one pass was almost 70% as opposed to 43% if placed in the proximal ICA (*p* = 0.021).

### Circle of Willis Anatomy

Figure 1 in the supplemental material presents the frequencies of anatomical variations in the CoW. The presence of an anterior communicating artery (ACoA) was the most common anatomical variant (76.5%, 153/200). Coexistence of an ACoA and a PCoA was observed in 22.5% of cases (45/200). The rate of mTICI 3 on the first pass did not differ significantly in cases of a coexisting ACoA and PCoA ipsilateral to the stroke side (60%, 27/45) as compared with all other anatomical variations combined (48.4%, 75/155) (*p* = 0.170).

Migration into a new territory occurred in six cases (five into the ACA and one into the posterior cerebral artery (PCA)). Four of these cases were treated with low-profile SR (for ≤ 3-mm vessels). In the logistic regression adjusted by CoW anatomy, the ICA/MCA ratio, intravenous thrombolysis, and intervention duration, the presence of an ACoA was an independent factor that afforded protection against migration into a new territory (reference: absent ACoA or contralateral A1 segment; OR 0.051; 95% CI, 0.004–0.667; *p* = 0.023). Furthermore, the risk of migration significantly increased by a factor of 3.579 (OR) with every 30 min of intervention time (95% CI, 1.558–8.224; *p* = 0.0027).

## Discussion

Single-pass mTICI 3 rates achieved by MT using an SR through a BGC ranged from 30% to a maximum of 70% depending on anatomical and interventional factors. Carotid elongation was the most potent biomarker for predicting an ineffective first pass (67% likelihood of non-mTICI 3 outcome). The more proximal or distal positioning of the BGC in the ICA initially appeared to be very promising, based on the univariate analysis results, but when adjusted to account for other factors, this factor no longer proved to be significant. Machine learning (decision tree) helped in interpreting the tangled relationship between carotid tortuosity and BGC positioning. Beyond the obvious impediments to advancing the BGC distally in cases of carotid tortuosity, we were able to identify a more decisive role of BGC location for therapy in cases of a straight carotid artery (no tortuosity). In this setting, the likelihood of complete first-pass recanalization with proximal BGC positioning was 43% compared with 69% for distal BGC positioning. Finally, the presence of communicating arteries that could hypothetically reduce the suction capacity of the BGC did not lower the first-pass mTICI 3 rate.

In agreement with previous studies, complete recanalization (mTICI 3, 62.5%) was commonly achieved in our series [18]. Nevertheless, the reported rates of complete recanalization using BGC have ranged from 15 to 63% [8, 19, 20]. We believe that these differences can be accounted for in part by differences in BGC use in the patients in these studies. That is, comparisons between BGC and other techniques are challenging when the use of flow arrest and concomitant aspiration during clot retrieval via the BGC cannot be guaranteed in all patients [7]. For example, in the ASTER trial (first-line aspiration versus first-line BGC + SR), the slight differences between the two treatment groups could perhaps be explained by the use of flow arrest alone without aspiration for SR thrombectomy, since there was no mention of concomitant aspiration for the BGC group in the published results [21] or in the study protocol [22].

In our study, using flow arrest and manual aspiration for retrieval in all cases, nearly 80% of all mTICI 3 outcomes were achieved with a single pass, with minimal gains in mTICI 3 outcomes after a second retrieval maneuver (13%). This highlights the extreme importance of removing a clot completely on the very first attempt. Consequently, identifying anatomical determining factors restricting the effectiveness of MT represents an initial step towards adapting the endovascular strategy and techniques to the anatomical conditions of patients for more efficient clot extraction. While one study did report target location in the distal ICA [8], specific BGC positioning for mechanical thrombectomy has seldom been described [5, 7, 18–20]. Our study revealed an efficacy gradient depending on the location of the BGC in the carotid artery, consistent with the results of Jeong et al. [13] (TICI 3 irrespective of passes in 67% of the distal BGC group versus 45% for the proximal BGC group). Nevertheless, in their analysis of 102 thrombectomies, Jeong et al. [13] merely described the relationship between artery elongation and catheter positioning as well as the relationship between elongation and recanalization, unsupported by any statistical test results. Our study, by contrast, additionally serves to elucidate the potential success of clot removal through a BGC in diverse circumstances. This would provide us with a basis for deciding, right from the onset, when to implement the BGC technique with a distal access catheter in order to enhance the chances of achieving first-pass mTICI 3 chances in specific anatomical conditions, without having to invest time fruitlessly in the conventional “three passes and change protocol”. From a femoral approach, it could be advantageous in difficult anatomical conditions to use a combination of proximal flow arrest and aspiration together with distal aspiration + SR. This combination of techniques derives benefit from all the positive aspects of each separate method used alone [23–26]. Moreover, we commonly add a long sheath over the BGC to get more support in cases with concomitant aortoiliac elongation.

Though simultaneous use of a BGC does seen to offer enhancement [23], first-choice direct aspiration through a distal access catheter has not yet demonstrated better rates of one-pass mTICI 3 than MT with SR through a BGC (concomitant flow arrest and aspiration) [18, 24]. Among other benefits, selecting the best patient-based clot removal approach from the very beginning leads to faster interventions. This could be one of the reasons why, in their meta-analysis, Texakalidis et al. [27] disclosed increased risk of symptomatic intracerebral hemorrhage when a combined approach (distal aspiration + SR) was used as the rescue therapy after failure of primary direct aspiration. Furthermore, such other options as removing the SR delivery microcatheter prior to retrieval with aspiration have, in vitro, exhibited an absolute gain of 0.3 mL/s in the water aspiration flow for an 8-French guide catheter and of nearly 1.5 mL/s for a 5-French distal access catheter [28]. We believe that this option is most probably unnecessary to improve aspiration capacity when larger-bore distal access catheters are used.

Something our experience has taught us is that, when performed without a distal access catheter, the removal of the SR delivery catheter before retrieval may lead to complications. Severe vasospasms or detachment of the retriever could occur because of an augmented in vivo resistance to retrieval when there is no support for the SR as recommended by the retriever manufacturers.

Finally, it has been suggested that reversed flow through the communicating arteries could reduce the suction effect, resulting in less effective clot extraction [7]. In our series, recanalization rates were not influenced by anatomical variations in the CoW. Migration into a new territory was infrequently observed (3%); however, when adjusted for intracranial anatomy, the presence of an ACoA was found to be a protective factor against migration. We believe that aspiration through the BGC under flow arrest could indeed be beneficial by reversing the flow in the communicating arteries. What matters is not the presence of an ACoA but the hemodynamic changes that can occur through the ACoA. Thanks to the pressure gradient influenced by aspiration under flow arrest, the blood from the contralateral carotid artery can readily flow through the ACoA to the treatment side, reversing the flow in the ipsilateral A1 segment. This inverted flow, now in the direction of the ICA, could therefore prevent the clot from entering the A1–A2 segments during retrieval from the MCA to the ICA [26, 29–31].

### Limitations

This study is a retrospective study from a single center. A significant limitation relates to the clinical outcomes, which were based on the NIHSS scores without consideration of long-term functional outcome, thus constraining the clinical value of the analysis. Furthermore, we did not collect data on the frequency of carotid dissections in relation to the position of the BGC for therapy, elongation, or the number of passes performed, and it could indeed be of interest to analyze that information together with the clinical outcomes. However, the objective of this study was focused on the factors influencing one-pass complete recanalization, currently the best angiographic outcome achievable by the fastest possible interventions. Recognizing the limitations of the technique in difficult anatomical constellations is a first step towards developing new techniques or combinations of techniques designed to enhance successful clot extraction on the first attempt.

## Conclusions

In terms of clinical practice, it can be first be concluded that the chances of achieving mTICI 3 decrease substantially after the first pass, and therefore, all efforts should be aimed at attaining successful clot removal on the first attempt. Secondly, carotid tortuosity is a biomarker for an unsuccessful first pass, and in such cases, modifying the method (for example, by using a distal access catheter + flow arrest + flow reversal) should be considered in an endeavor to enhance the likelihood of first-pass mTICI 3. By means of the decision tree, when the carotid artery is elongated, the BGC technique should always be optimized, irrespective of whether or not distal BGC positioning in the ICA is feasible. Thirdly, when the carotid artery is straight, the BGC should preferably be placed in the distal (subpetrosal) ICA, because the likelihood of mTICI 3 is highest under these conditions (no tortuosity and distal BGC).

## Electronic supplementary material

ESM 1 (DOCX 37 kb)

## Abbreviations

ACA: Anterior cerebral artery
ACoA: Anterior communicating artery
BGC: Balloon guide catheter
CCA: Common carotid artery
CI: Confidence interval
CoW: Circle of Willis
CTA: Computed tomography angiography
DSA: Digital subtraction angiography
ICA: Internal carotid artery
MT: Mechanical thrombectomy
mTICI: Modified Thrombolysis in Cerebral Infarction Scale
NIHSS: National Institutes of Health Stroke Scale
OR: Odds ratio
PCA: Posterior cerebral artery
PCoA: Posterior communicating artery
PH: Parenchymal hematoma
SR: Stent retriever
tPA: Tissue plasminogen activator
