# Supplementary material for: Predictors of Successful First-Pass Thrombectomy with a Balloon Guide Catheter: Results of a Decision Tree Analysis
Source: Transl Stroke Res. 2020 May 23;11(5):900–9. doi: 10.1007/s12975-020-00784-2 (PMC7496051; doi:10.1007/s12975-020-00784-2)
Supplement: Supplementary file 1 — (DOCX 37 kb) [file 12975_2020_784_MOESM1_ESM.docx]

**Supplemental Material**

***Table 1*:** List of stent retrievers (SRs) used, grouped by number of interventions in which a single SR or multiple SRs were required per intervention.

| Type | Single type/intervention | Multiple SRs/intervention | Total SRs used |
| --- | --- | --- | --- |
| Preset 4 | 124 | 20 | 144 |
| Preset 4 LT | 1 | 1 | 2 |
| Preset 6 | 26 | 11 | 37 |
| Preset 3 | 6 | 5 | 11 |
| Embotrap | 6 | 1 | 7 |
| Trevo 4 | 6 | 0 | 6 |
| Solitaire 4 | 2 | 1 | 3 |
| Solitaire 6 | 1 | 1 | 2 |
| 3D separator | 0 | 1 | 1 |
| Eric 3 | 0 | 1 | 1 |
| Eric 4 | 1 | 5 | 6 |
| Eric 6 | 1 | 4 | 5 |
| Mindframe Capture 3 | 0 | 2 | 2 |
| Catchmini | 0 | 1 | 1 |
| Total SRs, n= | 174 | 54 | 228 |

In order of appearance: Preset SR (Phenox, Bochum, Germany); Embotrap (Cerenovus, Galway, Ireland); Trevo SR (Stryker Neurovascular, Fremont, California, USA); Solitaire SR (EV3 Neurovascular, Irvine, CA, USA); 3D separator (Penumbra Inc, Alameda, CA, USA), Eric SR (Microvention, Tustin, CA, USA); Mindframe Capture 3 (Medtronic, Minneapolis, MN, USA); and Catchmini (Balt, Montmorency, France).

***Figure 1:*** Association of anatomical variants, with illustrations representing anatomical variations for a right-sided occlusion

|  |  |  | | Posterior communicating artery | | | | |  |
| --- | --- | --- | --- | --- | --- | --- | --- | --- | --- |
| Anterior cerebral artery |  | |  | | **A** | **B** | **C** |  | |
|  |  | |  | | 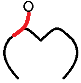 | 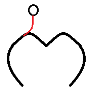 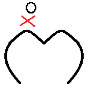 | 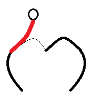 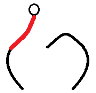 | Total | |
|  | **A** | | 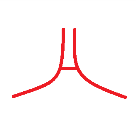 | | 45 (22.5) | 92 (46) | 16 (8) | 153 (76.5) | |
|  | **B** | | 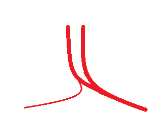 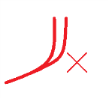 | | 2 (1) | 4 (2) | 2 (1) | 8 (4) | |
|  | **C** | | 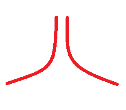 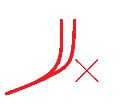 | | 7 (3.5) | 25 (12.5) | 7 (3.5) | 39 (19.5) | |
|  |  | | Total | | 54 (27) | 121(60.5) | 25 (12.5) | 200 | |

Units in parentheses are percentages. PCoA, posterior communicating artery; ACoA, anterior communicating artery; A1, A1 segment of the anterior cerebral artery (ACA)

ACoA anatomical variations: (A) Bilateral A1 segment plus ACoA; (B) Ipsilateral A1 segment absent or hypoplastic; (C) No ACoA or contralateral A1 absent or hypoplastic

PCoA anatomical variations: (A) PCoA; (B) PCoA absent or hypoplastic; (C) Fetal type
